# Brain reactivity to emotional stimuli in women with premenstrual dysphoric disorder and related personality characteristics

**DOI:** 10.18632/aging.203363

**Published:** 2021-08-04

**Authors:** Mingzhou Gao, Mingqi Qiao, Li An, Guangbin Wang, Jieqiong Wang, Chunhong Song, Fengqin Wei, Yanhong Yu, Tao Gong, Dongmei Gao

**Affiliations:** 1 College of Traditional Chinese Medicine, Shandong University of Traditional Chinese Medicine, Jinan, Shandong Province, China; 2 Research and Innovation Team of Emotional Diseases and Syndromes in Shandong University of Traditional Chinese Medicine, Jinan, Shandong Province, China; 3 Key Laboratory of Traditional Chinese Medicine Classical Theory, Ministry of Education, Shandong University of Traditional Chinese Medicine, Jinan, Shandong Province, China; 4 Jinan Central Hospital, Jinan, Shandong Province, China; 5 Department of Radiology, Shandong Medical Imaging Research Institute, Jinan, Shandong Province, China; 6 Department of Laboratory Animal Center, Central Hospital Affiliated to Shandong First Medical University, Jinan, Shandong Province, China

**Keywords:** premenstrual dysphoric disorder, anger, depression, eysenck personality questionnaire, emotion regulation

## Abstract

Aims: Premenstrual dysphoric disorder (PMDD) is a psychiatric condition that is associated with the menstrual cycle. Elucidation of the neural regulation mechanisms of brain reactivity to emotional stimuli among women with PMDD may inform PMDD treatment.

Methods: Eighty-six women (42 PMDD, 44 healthy controls) were allocated into two groups (anger-induced group: 23 PMDD vs. 23 controls; depression-induced group: 19 PMDD vs. 21 controls). During the luteal phases of the menstrual cycle, all the women were subjected to functional magnetic resonance imaging (fMRI). fMRI resting-state scans were performed before and after the study participants had performed an emotional stimuli task. After the emotional stimuli task, emotional status of the participants were evaluated by Self-Rating Depression Scales (SDS) and Trait Anger Expression Inventory–II (STAXI-II). In addition, all the participants were requested to complete the Eysenck Personality Questionnaire (EPQ) and the Twenty-Item Toronto Alexithymia Scale (TAS-20).

Results: Compared to healthy controls, all women with PMDD exhibited significantly high scores in Tas-20 (p<0.001), higher neuroticism and psychoticism scores as well as significantly low extraversion and social desirability scores (p<0.05). Compared to the controls, f-MRI revealed that PMDD women had elevated ReHo in the middle frontal gyrus (BA10), temporal lobe (BA42), left cerebellum (BA37), as well as decreased activation in the precuneus (BA7), superior frontal gyrus (BA8), lobulus paracentralis (BA6), and right cerebellum (BA48) (p<0.05). Moreover, depression stimuli showed that women with PMDD had elevated ReHo levels in the middle frontal gyrus (BA11), the middle gyrus (BA47) and in the cingulate gyrus (BA23) vs. healthy controls (p<0.05).

Conclusions: Women with more neuroticism and psychoticism, less extraversion and social desirability tend to report PMDD symptoms. Women with this condition experience difficulties in regulating emotions during the luteal phase of the menstrual cycle. Abnormal ReHo levels in the precuneus, superior frontal gyrus, lobulus paracentralis, and right cerebellum may contribute to anger dysregulation. Hypoactivation in the middle frontal gyrus, the middle gyrus and the cingulate gyrus may be generally associated with depression dysregulation in PMDD.

## INTRODUCTION

Premenstrual dysphoric disorder (PMDD), a severe form of premenstrual syndrome (PMS), is characterized by significant premenstrual mood disturbances, often with “a cluster of affective, behavioral and somatic symptoms” [1]. Based on the latest diagnostic criteria, PMDD was classified as a subclass of depressive disorders in the Diagnostic and Statistical Manual of Mental Disorders (DSM–5) in 2013 [2]. According to DSM-5, 3-8% of women in the reproductive age have PMDD [3], which causes a severe decrease in the quality of life and psychological problems [4, 5].

Depression, anxiety, and irritability are the three most evaluated PMDD symptoms [6]. Emotional problems constitute most of the PMDD symptoms, therefore, it has been postulated that women with PMDD experience greater difficulties with emotion regulation. Emotional regulation is the ability to identify the emotions a person feels, and how those emotions are experienced, expressed and regulated [7]. The mechanisms through which PMDD leads to emotional dysregulation have not been elucidated. However, it has been postulated that central nervous system (CNS) sensitivity to reproductive hormones, such as progesterone and allopregnanolone, genetic factors, as well as psychosocial factors, such as trauma history or emotional and physical abuse may be contribute to emotional dysregulation [8]. Imaging studies have reported differences in brain structure and function between women with and without PMS/PMDD [9–11]. These findings regarding abnormal activities of the brain in PMDD may be potential key factors for the occurrence of it [12].

Functional magnetic resonance imaging (fMRI) based on blood-oxygen-level dependent (BOLD) techniques has widely been used to study functional activities and cognitive behaviours of the brain in response to induced stimuli with tasks, that is, task fMRI (tfMRI) or without tasks, that is, resting state fMRI (rsfMRI) [13, 14]. Structurally, women with PMDD have been shown to exhibit greater grey matter density in the hippocampal cortex and lower grey matter density in the parahippocampal cortex [15]. Functionally, women with PMDD have elevated amygdala and suppressed ventral striatum responses to negative stimuli during the luteal phase [16]. Moreover, during the menstrual cycle, women with PMDD exhibit lower activations of the perennial anterior cingulate and the ventromedial prefrontal cortex [17]. A recent study reported that, compared to healthy controls, women with PMDD have different intrinsic network dynamics in the brain [18].

Based on the above findings, this study aimed at investigating differences in brain reactions when women with PMDD and healthy controls (HCs) are subjected to emotional stimuli.

## MATERIALS AND METHODS

### Ethical statement

After being informed of the procedures in the study as well as the significance of the study, participants were asked to sign an informed consent form before inclusion in the study. Ethical approval was obtained from the Medicine Ethics Committee of the First Affiliated Hospital of Shandong University of Traditional Chinese Medicine, Shandong, China. All research procedures were performed in accordance with the Declaration of Helsinki.

### Study participants

We performed an epidemiological survey whereby a total of 868 questionnaires were distributed among women in universities in Jinan, Shandong, China. A total of 786 questionnaires were recovered for collecting demographic data. Based on the DSM-5 criteria, 46 women were diagnosed with PMDD [19]. A total of 46 healthy volunteers with no history of mental illnesses and who were in good physical health were recruited from universities through newspaper, online and leaflet advertising. All participants with PMDD, in accordance with the random number table, were randomly allocated into the anger-induced group (23 PMDD vs. 23 controls) and the depression-induced group (19 PMDD vs. 21 controls). Data for four PMDD participants and two controls in the depression-induced group were excluded from the final analysis because two participants failed to experience anger while four participants had correctly guessed the purpose of the experiment before it had started.

### Inclusion criteria for PMDD

The inclusion criteria for the PMDD group were: i. Women who met the DSM-5 PMDD diagnostic criteria; ii. Female, 18–45 years old, ethnic origin was not a consideration; iii. Women with a normal menstrual cycle (differences in the range of duration of menstrual flow ≤ 3 d), cycle 21–35 d; iv. Women who had demonstrated an understanding of the purpose of this study and were willing to volunteer; v. Women without major diseases including cardiac, liver, and kidney diseases as well as brain tumors or other brain diseases; vi. Women without a history of drug abuse.

### Inclusion criteria for HC

The inclusion criteria for HC were: i. Female, 20–25 years old, right-handed, volunteer college students; ii. Regarding consciousness and independent judgment, we included women who demonstrated an understanding of the purpose of this study and who were willing to volunteer; iii. Women with normal visual acuity with or without correction; iv. Lack of metal objects in the body (including pacemakers, metal dental materials, and braces among others); v. Healthy individuals without frequent headaches, dizziness, seizures, or other neurological diseases; vi. Women in good mental states, with good sleep quality and appetite.

### Exclusion criteria for all study participants

Exclusion criteria for the study were: i. mental illness or women with previous histories of mental illness; ii. Serious physical illness; iii. Women with a history of drug abuse (including three months of treatment with PMDD drugs); iv. Those with hematological diseases; v. Pregnant or lactating women; vi. Aphasia, disturbance of consciousness, dementia, and other circumstances such that participants could not cooperate with the examiner; vii. Months of unilateral ovariectomy or abortion, taking contraceptives; viii. Head movement more than 3 mm, in any direction, and more than 1° during motion correction.

Participants were terminated from the study if they: i. Exhibited symptoms requiring emergency treatment, thereby interfering with clinical study of the case; ii. Were unable to adhere to the study; and iii. Became pregnant.

### Personality measures

Personality characteristics were determined using the Eysenck Personality Questionnaire (EPQ), which is an 88-item questionnaire that measures the four personality dimensions (21 items for extraversion, 23 items for psychoticism, 24 items for neuroticism, and 20 items for lying/social desirability) [20]. Scores were summed and converted into T scores using the equation: (T=50+10*(X-M)/SD).

### Emotional evaluation

Emotional regulation was measured using the Twenty-Item Toronto Alexithymia Scale (TAS-20), which was used to test participants’ self-inability to identify and describe emotions before f-MRI. Emotional evaluation after induction was performed using the Self-Rating Depression Scale (SDS) while anger was measured using the State-Trait Anger Expression Inventory-2 (STAXI-2) created by Spielberger. The State–Trait Anger Expression Inventory–II (STAXI-II) is a psychometric assessment tool that is used to measure anger experience, expression, and control in research and in clinical settings.

### Experimental paradigm

Participants in the two PMDD groups and their related HC groups were subjected to fMRI examination during the late luteal phase (ranging from 1 to 5 d before menstruation). To confirm the relatively stable and low levels of endogenous cortisol and oestradiol, the scan tests were performed between 19:00 and 22:00 pm [21]. To verify menstrual cycle stages, we obtained self-reports regarding when menstruation started and combined this information with primary gynaecological examinations.

**Figure 1:**
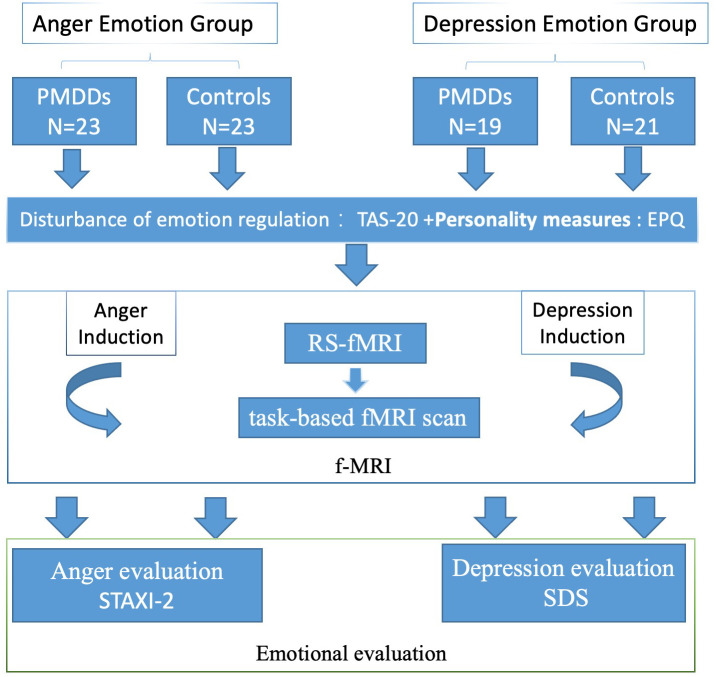
Schematic presentation of the experimental procedure through which participants watched various images in the anger- and depression-induction stages.

In the entire experimental procedure, anger and depression were induced in different groups (Figure 1). Each study participant was subjected to an fMRI scan composed of 6 min of 3D structure image scanning and 8 min of resting-state (RS-fMRI) scanning followed by a task-based fMRI scan during which emotion images were presented. During the RS-fMRI scan, each study participant was instructed to keep her eyes closed, not to think about anything and to stay awake.

During task-based fMRI scans, participants had an option of keeping their eyes open and view negative (anger for anger-induced group and depression for depression-induced group). Neutral (NEU) emotion images were selected from the International Affective Picture System [22] based on our previous studies. The task-based fMRI scan was performed in two runs, with one set of neutral and negative images (6 images each) presented in each run. The first run consisted of a 30-sec presentation of anger images (each image was presented for 5 s, 6 images in a block) followed by a 30-sec presentation of neutral images (each image was presented for 5 s, 6 images in a block). The second run was performed in the opposite order.

Stimulation images were presented using a brain function audio-visual stimulation system (SAMRTEC SA-9900; Shenzhen Meide Medical Electronics Technology Co., Ltd). This system composed of a general console, cabinets, mirrors, vinyl screens, and liquid crystal displays among others. Based on experimental requirements, using a projector, study participants viewed clear visual images as selected by experimenters. The experimental visual stimulation system was programmed with Eprime. Both groups were shown negative emotional pictures and neutral emotional pictures as stimuli for the task. Negative emotions were induced by viewing the pictures.

After the scan, subjective reports of picture-evoked emotional effects were evaluated using the self-rating depression scale (SDS) and the emotional statement and guidance language implementation checklist. Participants’ subjective feelings were evaluated using the visual scale test, which ranged from 0 (no feeling) to 8 (very strong feeling) points. Higher scores implied that participants experienced higher emotional strengths. Participants were asked to carefully assess their emotional intensities.

### Data acquisition

The fMRI imaging device is a magnetic device manufactured by the Philips Company, Netherlands. It consists of a 3.0t TX superconducting MR instrument, and an eight-channel phase-control front ring. FMRI images were obtained using a 3.0-T MR scanner equipped with a prototype fast gradient system for echo-planar imaging (EPI) at the Institute of Medical Imaging of Shandong. Functional images were obtained using an echo planar imaging sequence with the following parameters: TE = 35 ms, TR = 2000 ms, slice thickness = 4 mm, gap = 1 mm, flip angle = 90°, FOV = 24 cm, and in-plane resolution = 64 × 64. The resting-state session lasted 6 min, during which participants were instructed not to; move, think systematically or to fall asleep. In addition, a T1-weighted sagittal three-dimensional magnetization-prepared rapid gradient echo (MP-RAGE) sequence was acquired with the following parameters: 144 slices, TR = 2300 ms, TE = 3.39 ms, slice thickness = 1 mm, flip angle = 7°, inversion time = 1100 ms, FOV = 200 × 256 mm^2^, and in-plane resolution = 200 × 256.

### fMRI data analysis

Functional MRI data were pre-processed using Statistical Parametric Mapping (SPM8) [23]. The first 3 volumes of functional images were discarded because of signal equilibrium and to allow participants to adapt to the scanning noise. All images were time-shifted so that the slices were temporally aligned. Then, images were realigned, after which we verified that all participants had moved no more than 3 mm in the translational dimension or 3° in the rotational dimension. Anatomical images were spatially normalized to the Montreal Neurological Institute (MNI) template. Normalization parameters were applied to functional images. Images were smoothed using a Gaussian filter with a full width of 8 mm at half maximum.

All images were time-shifted so that slices were temporally aligned. Then, images were realigned, after which we verified that all participants had moved no more than 3 mm in the translational dimension or 3° in the rotational dimension. Then, images were co-registered with anatomical images, which were segmented into grey matter and white matter. Anatomical images were spatially normalized to the Montreal Neurological Institute (MNI) template, and normalization parameters were applied to the functional images. Images were smoothed using a Gaussian filter with a full width of 8 mm at half maximum. After further pre-processing, which included the removal of linear trend and temporal bandpass filtering (0.01-0.08 Hz), regional homogeneity (regional homogeneity, ReHo) was determined using the Resting-State fMRI Data Analysis Toolkit (REST, by Song et al., http://www.restfmri.net).

### Statistical analysis

To examine the personality and effects of the two emotion induction procedures on participants’ subjective feelings of anger, depression as well as their positive and negative emotions, we performed unpaired T tests to compare emotional scores for PMDD vs. controls in the two emotion groups. All statistical tests were performed using the SPSS 25.0 software. Continuous variables are expressed as mean ± standard deviation. p≤0.05 was considered statistically significant.

Functional MRI data for our study were modelled in a generalized linear mode (GLM) using SPM8. The data conformed to statistical requirements. Group-level statistical analyses were performed using a random-effects model in SPM8. Two-sample t-tests were performed on individual activation maps of the two groups with small volume correction for one sample result data. Data were corrected for multiple comparisons using the Monte Carlo simulation (see program AlphaSim by B.D. Ward, http://afni.nimh.nih.gov/pub/dist/doc/manual/AlphaSim.pdf). Significant between-group differences met the criteria of uncorrected p < 0.01 at the voxel level and cluster size > 40 voxels, corresponding to a corrected p < 0.05. Then, to examine the altered activation difference, one-sample t-tests were performed on the individual activation maps of between-group peak voxels in the two groups, with a significance criterion of p < 0.05 in the SPSS 25.0 software package. Finally, we evaluated the dissociable anomaly of activation patterns between two groups in the whole brain using the criterion of corrected p < 0.05 for voxel level and cluster size > 389 voxels. The alpha for all significant results was two-tailed, except where indicated.

### Data

The data analyzed in this study are available from the authors upon reasonable request.

### Ethical standards

This study was ethically approved by the Medicine Ethics Committee of the First Affiliated Hospital of Shandong University of Traditional Chinese Medicine, Shandong, China. All research procedures were conducted in accordance with the Declaration of Helsinki.

## RESULTS

### Participant characteristics

There were no significant differences in age, menstruation (days), menophania (years), or length of menstrual cycle (days) between the anger- or depression-induced groups.

### Personality characteristics and emotion regulation

**Figure 2:**
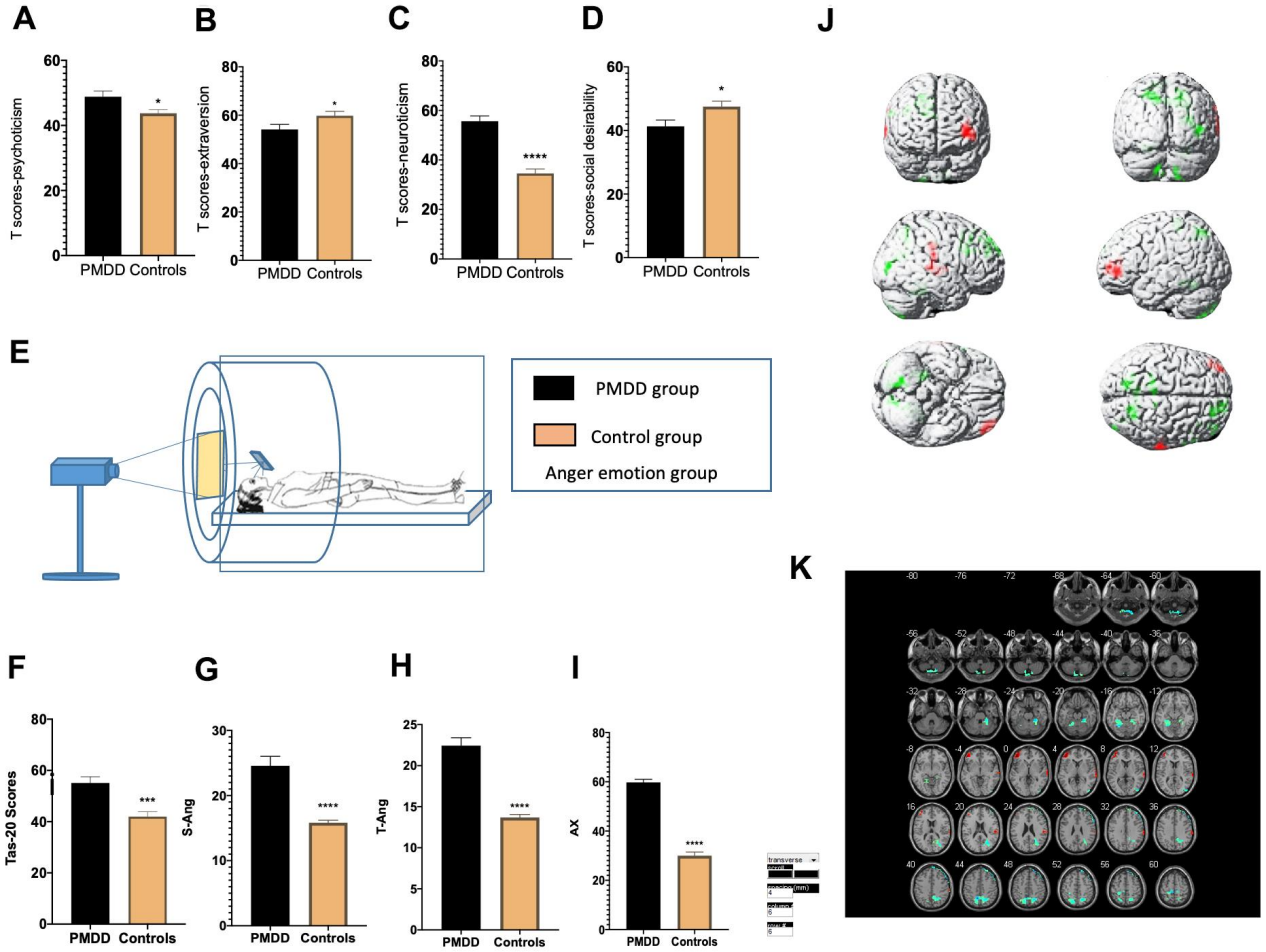
**Comparisons of emotional changes and brain reactivity to emotional stimuli among PMDD vs. control study participants (PMDD=23, controls=23) of the anger-induced group.** (**A**–**D**) Extraversion, psychoticism, neuroticism, and social desirability T-scores. (**E**) Schematic presentation of the study participants in the experiment. (**F**) TAS-20 scores for both groups. (**G**–**I**) Anger emotions in the anger-induced group. (**J**) Illustration of activations in various brain areas. Compared to the HC group, the PMDD group exhibited increased activation, mainly in the middle frontal gyrus (BA10), temporal lobe (BA42), main part of left cerebellum (BA37), as well as decreased activation in the precuneus (BA7), superior frontal gyrus (BA8), paracentral lobule (BA6), and right cerebellum (BA48). (**K**) PMDD group and HC group Anger mood Subtract Neutral Frontal Mid-Back Picture Condition T-test activation Differential area; p < 0.05, cluster size > 389 warm (T value positive) represents PMDD group higher than the HC group, cool (T negative) on behalf of the normal group than the patient group.

**Figure 3:**
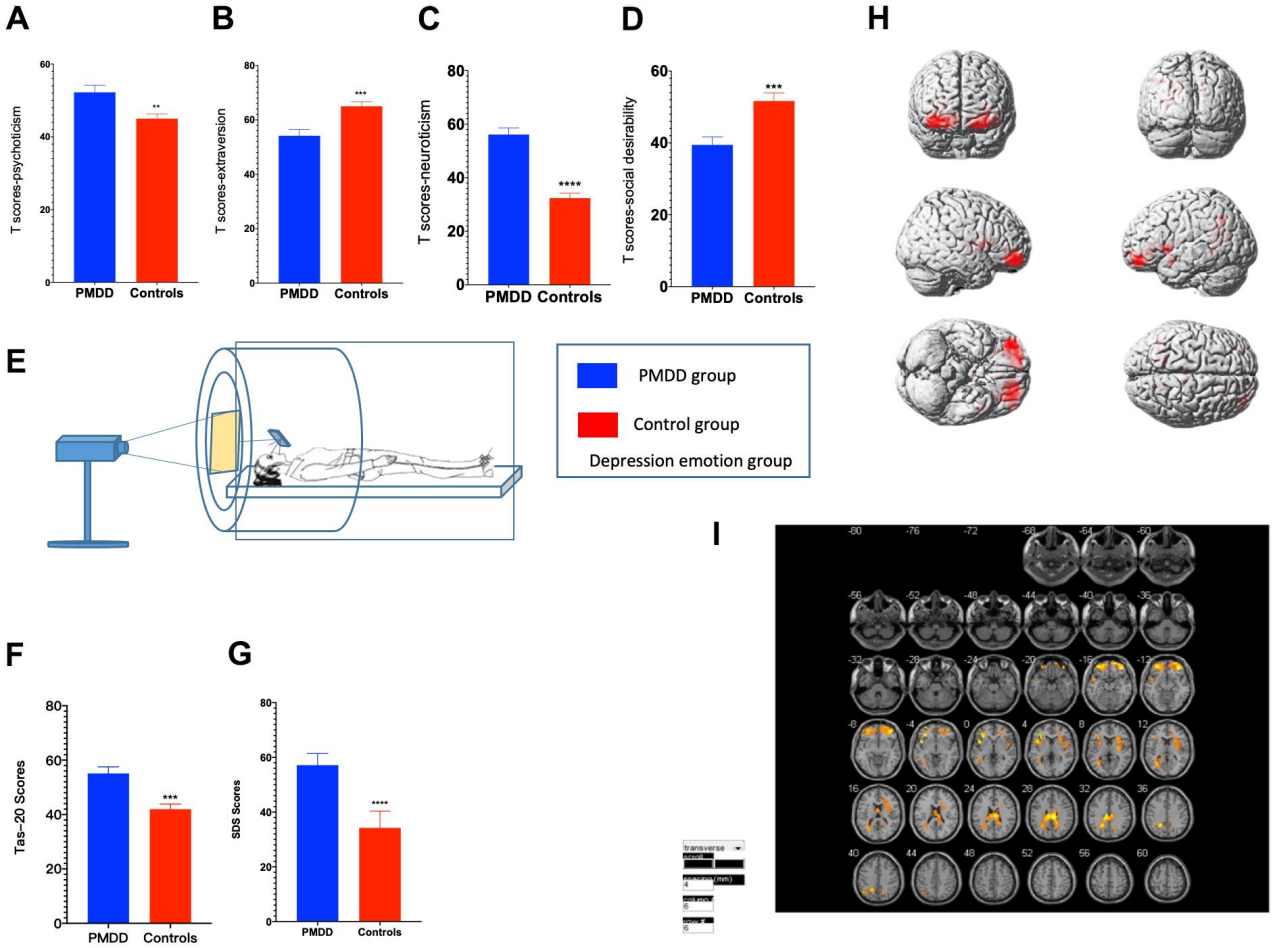
**Comparisons of emotional changes and brain reactivity to emotional stimuli among PMDD and control study participants (PMDD=19, Controls=21) of the depression-induced group.** (**A**–**D**) Extraversion, psychoticism, neuroticism, and social desirability T-scores. (**E**) Schematic presentation of participants in the experiment. (**F**) TAS-20 scores for both groups. (**G**) Depression emotions of participants in the depression-induced group. (**H**) Illustration of activations in various brain areas: the middle frontal gyrus, middle gyrus and cingulate gyrus for PMDD and HC groups. (**I**) PMDD and HC groups Depression mood Subtract Neutral Frontal Mid-Back Picture Condition T-test activation Differential area; p < 0.05, cluster size > 389 warm (T value positive) represents PMDD group higher than the HC group, cool (T negative) on behalf of the normal group than the patient group.

In the anger-induced group, women with PMDD exhibited higher Tas-20 scores (p=0.0001), higher neuroticism and psychoticism T-scores (p<0.0001 and p=0.0204, respectively) as well as lower extraversion and social desirability T-scores (p=0.0477 and p=0.0222, respectively). In the depression-induced group, women with PMDD exhibited higher Tas-20 scores (p=0.0001), higher neuroticism and psychoticism T-scores (p<0.0001 and p=0.0027, respectively), as well as lower extraversion and social desirability T-scores (p=0.0004 and p=0.0005, respectively) (Figures 2A–2D, 2F, 3A–3D, 3F).

### Emotional evaluation

For anger induction, both groups exhibited a strong degree of anger. However, compared to the controls, women with PMDD exhibited higher AX, S-Ang and T-Ang scores (p<0.0001, p<0.0001 and p<0.0001, respectively) when evaluated using STAXI-2. These findings imply a higher level of anger. For depression induction, both groups showed a strong degree of depression and anxiety. However, compared to the controls, women with PMDD exhibited higher SDS scores (p<0.0001), implying a higher level of depression in this group (Figures 2G–2I, 3G).

### tfMRI

tfMRI was used to study brain functional activities and cognitive behaviors based on a task with an induced stimulus. Compared to the HC group, after anger induction, the PMDD group mainly exhibited elevated activation in the middle frontal gyrus (BA10), temporal lobe (BA42), and left cerebellum (BA37) as well as decreased activation in the precuneus (BA7), superior frontal gyrus (BA8), lobulus paracentralis (BA6), and in the right cerebellum (BA48) (p<0.05). After depression-induction, compared to the HC group, the PMDD group exhibited increased activation in the middle frontal gyrus (BA11), the middle gyrus (BA47) and in the cingulate gyrus (BA23) based on BOLD-fMRI (p < 0.05) (Figures 2J, 2K, 3H, 3I and Supplementary Tables 1, 2).

## DISCUSSION

Clinically, difficulties in regulating emotions are linked to core PMDD symptoms [18]. In this study, tas-20 scales indicated that all PMDD women had dysregulated feelings. Personality traits reflect people’s characteristic patterns of thoughts, feelings, and behaviours. The EPQ is a questionnaire used in psychology to assess personality traits of an individual. The questionnaire was initially devised by psychologists Hans Jürgen Eysenck and Sybil B. G. Eysenck [24]. According to the EPQ, compared to controls, PMS patients score significantly higher with regard to somatic anxiety, muscular tension, indirect aggression, verbal aggression and neuroticism and lower with regards to socialization [25]. Impaired cognitive functions are key in defining PMDD. In this study, we adopted EPQ to investigate susceptible traits. Compared to HCs, neuroticism and psychoticism scores were higher while extraversion and lying scores were lower in PMDD patients, implying that there are certain personality tendencies in PMDD.

Eysenck’s biological model of personality suggests that a quitting behaviour is strongly correlated with extraversion scores. In other words, extroverted individuals tend not to persevere when solving boring, frustrating problems [26]. YA Zhang found that different personalities lead to different PMS symptoms and coping styles [27]. A recent study evaluating recurrent depression in recurrent brief depression (RBD) revealed that there were significant differences in the distribution of neuroticism (N) and extraversion (E) scores between patients and controls. Anxiety groups exhibited high N scores while RBD patients were found to have low E scores [28]. Most women with menstrual disorders are characterized by neuroticism, which can affect their quality of life [29]. Neurotic women are less vulnerable to negative emotions during their mid-late luteal phase than during other phases. Sensitive responses of neurotic women to fluctuations in progesterone levels during menstrual cycles may be among the reasons accounting for their mood changes [30].

According to Hans Eysenck’s theory, personality traits have a close relationship with brain activity [31]. In this study, we found that compared to healthy controls, after depression induction, brain activities were enhanced in the middle frontal gyrus, middle gyrus and in the cingulate gyrus of PMDD women. Moreover, after anger induction, PMDD women exhibited increased activations mainly in the middle frontal gyrus (BA10), temporal lobe (BA42), left cerebellum (BA37), as well as decreased activations in the precuneus (BA7), superior frontal gyrus (BA8), lobulus paracentralis (BA6), and right cerebellum (BA48). Furthermore, our findings indicate that brain function abnormalities occur in patients before menstruation, which affects their emotional capacities as well as cognitive abilities. Our findings are consistent with those of previous studies, the only difference is that previous studies did not perform in-depth research on specific PMDD emotions [10, 32].

Clinical and neurological studies have not conclusively determined whether the frontal lobe plays a key role in emotional processing. Some studies have reported that frontal lobe lesions can lead to changes in patient moods [33, 34]. In particular, associative fibers in the frontal part of the frontal lobe are closely associated with mental activities [35, 36]. This series of direct or indirect neural connections are the anatomical bases for the regulation of physiological and psychological functions of the prefrontal cortex [37]. For PMDD patients during the luteal phase, emotional responses to negative emotion pictures were significantly reduced. This indicates that positive emotional adjustments for patients were weakened while negative emotional regulations were increased during the premenstrual period [38]. Our results are consistent with those of Gingnell and Baller who used PET or fMRI to investigate CNS activity and confirmed that prefrontal cortex reactivity in PMDD patients was significantly enhanced when compared to that of healthy controls [21, 39].

The cerebellum processes a wide range of behavioral effects, including pain, emotional, and administrative functions [40, 41]. In the luteal phase, PMS patients show increased cerebellar activation, especially in the cerebellar vermis. Enhanced cerebellar activity is associated with emotional deterioration [42, 43]. These findings imply that the cerebellar nucleus, which is associated with other mood disorders, may also be involved in PMDD patients. The involved primary regions are the top of the cerebellum midline and cerebellar vermis [44, 45]. We found that brain regions presented in the experiment were more specific than in previous studies, further demonstrating that the occurrence of PMDD is associated with the cerebellum.

The frontal lobe is located in the front of the brain and it includes four main gyri. The frontal lobe is the most developmentally advanced brain structure, possessing different emotional processing functions. The frontal cortex edge affects individual decision making and emotional regulation [33]. It also affects almost all psychological functions to which emotional control of an individual are inextricably linked. As an important part of brain emotional control, the cingulate gyrus may also play a key regulatory role in individual cognitive functions, emotional regulation and so on. The anterior cingulate gyrus also plays an important role in measuring both external and self-expected matches [46]. When the cingulate gyrus is damaged, human implementation of cognitive, emotional and other brain functions is dysregulated, which triggers individual indifference, attention disorders, autonomic dysfunction, emotional instability and other clinical symptoms [47]. Abnormal changes in the cingulate gyrus are associated with individual mental anxiety [48, 49]. Comasco et al. [50] found out anterior cingulate cortex activation by emotional stimulation in PMDD patients.

### Strengths and limitations

This study has some strengths, for instance, the sample size was prospectively determined and was bigger than that of existing published studies, which evaluated brain activity in women with PMDD [18, 51, 52]. Our findings inform on treatment avenues for PMDD. A major limitation of this study was that study participants were obtained from universities and lacked women of other ages, which limits the application of our findings.

## CONCLUSIONS

Neuroticism and psychoticism are susceptible traits of PMDD patients and are associated with brain reactivity to emotional stimuli. Upon exposure to depressive stimuli, we found increased functions of the middle frontal gyrus, the middle gyrus and the cingulate gyrus in PMDD women. Upon exposure to anger stimuli, the frontal lobe (especially the upper, middle, and central lobule), parietal lobes (mainly the precuneus), temporal lobe, and cerebellum (mainly the left and right cerebellum) were activated in PMDD women. More studies using larger samples are needed to confirm our findings and to identify neural circuit mechanisms to emotion regulation.

## Supplementary Material

Supplementary Tables
