# Supplementary material for: Brain reactivity to emotional stimuli in women with premenstrual dysphoric disorder and related personality characteristics
Source: Aging (Albany NY). 2021 Aug 4;13(15):19529–41. doi: 10.18632/aging.203363 (PMC8386568; doi:10.18632/aging.203363)
Supplement: Supplementary Tables [file aging-13-203363-s001.pdf]

## SUPPLEMENTARY TABLES

**Supplementary Table 1. PMDD group and HC group depression mood subtract neutral frontal mid-back picture condition t-test activation differential area.**

| Central position | MNI coordinate | | | Voxel | t | BA |
|---|---|---|---|---|---|---|
| | X | Y | Z | | | |
| the middle frontal gyrus | -30 | 48 | -12 | 588 | 3.321 | 11 |
| middle gyrus | 39 | 42 | -15 | 957 | 3.963 | 47 |
| cingulate gyrus | -6 | -24 | 30 | 1072 | 4.154 | 23 |

Note: PMDD, premenstrual dysphoric disorder; HC, healthy control; MNI, Montreal Neurological Institute; BA, Brodmann area.

**Supplementary Table 2. PMDD group and HC group anger mood subtract neutral frontal mid-back picture condition t-test activation differential area.**

| Brain region | MNI coordinates | | | Voxel | *t* | BA |
|---|---|---|---|---|---|---|
| | X | Y | Z | | | |
| Superior frontal gyrus | 24 | 42 | 51 | 225 | −3.4197 | 8 |
| Middle frontal gyrus | −42 | 45 | 0 | 188 | 2.8693 | 10 |
| paracentral lobule | −6 | −33 | 60 | 142 | −3.1293 | 6 |
| Precuneus | −9 | −57 | 45 | 730 | −3.6001 | 7 |
| Temporal lobe | 72 | −30 | 6 | 138 | 2.4814 | 42 |
| Right cerebellum | 24 | −39 | −21 | 185 | −2.8195 | 48 |
| Left cerebellum | −21 | −57 | −18 | 200 | 2.9857 | 37 |

Note: PMDD, premenstrual dysphoric disorder; HC, healthy control; MNI, Montreal Neurological Institute; BA, Brodmann area.
